# From Data Processing to Mental Organs: An Interdisciplinary Path to Cognitive Neuroscience**

**DOI:** 10.4103/0973-1229.77438

**Published:** 2011

**Authors:** Manoj Patharkar

**Affiliations:** * *Assistant Professor, Joshi Bedekar College, Thane, India.*; ** *Revised and peer reviewed version of a paper which won the Mens Sana Monographs Young Researcher Award in the Young Researchers Award Session at an International Seminar on Mind, Brain, and Consciousness, Thane College Campus, Thane, India, January 13-15, 2010.*

**Keywords:** *Chomsky*, *Computer system*, *Human brain*, *Mental Organs*, *Mind*, *Language faculty*

## Abstract

Human brain is a highly evolved coordinating mechanism in the species Homo sapiens. It is only in the last 100 years that extensive knowledge of the intricate structure and complex functioning of the human brain has been acquired, though a lot is yet to be known. However, from the beginning of civilisation, people have been conscious of a ‘mind’ which has been considered the origin of all scientific and cultural development. Philosophers have discussed at length the various attributes of consciousness. At the same time, most of the philosophical or scientific frameworks have directly or indirectly implied mind-body duality. It is now imperative that we develop an integrated approach to understand the interconnection between mind and consciousness on one hand and brain on the other. This paper begins with the proposition that the structure of the brain is analogous, at least to certain extent, to that of the computer system. Of course, it is much more sophisticated and complex. The second proposition is that the Chomskyean concept of ‘mental organs’ is a good working hypothesis that tries to characterise this complexity in terms of an innate cognitive framework. By following this dual approach, brain as a data processing system and brain as a superstructure of intricately linked mental organs, we can move toward a better understanding of ‘mind’ within the framework of empirical science. The one ‘mental organ’ studied extensively in Chomskyean terms is ‘language faculty’ which is unique in its relation to brain, mind and consciousness.

## Introduction

The aim of this paper is to suggest that by following a dual approach, brain as a data processing system and brain as a superstructure of intricately linked mental organs, we can move toward a better understanding of ‘mind’ within the framework of empirical science. It begins with the proposition that the structure of the brain is analogous, at least to certain extent, to that of the computer system. Of course, it is much more sophisticated and complex. The second proposition is that the Chomskyean concept of ‘mental organs’ is a good working hypothesis that tries to characterise this complexity in terms of an innate cognitive framework.

This paper consists of the following two parts: (1) Analogy between brain and computer system; and (2) Noam Chomsky’s Idea of ‘mental organ.’

Human brain is a highly evolved coordinating mechanism in the species Homo sapiens. It is only in the last 100 years that extensive knowledge of the intricate structure and complex functioning of the human brain has been acquired, though a lot is yet to be known. However, from the beginning of civilisation, people have been conscious of a ‘mind’ which has been considered the origin of all scientific and cultural development. Philosophers have discussed at length the various attributes of consciousness. Consciousness is being conscious of the outer world as well as of itself. Human being is conscious of his own consciousness.

Most of the philosophical or scientific frameworks have directly or indirectly implied mind-body duality. It is now imperative that we develop an integrated approach to understand the interconnection between mind and consciousness on one hand and brain on the other. One way of doing this is to begin with studying the analogy between human brain and a computer system.

## Brain and the Computer

Before exploring the analogy, it must be made clear that the analogy is a beginning, an orientation. It is pursued to notice the similarities in order to understand the distinctive features of the human brain. The main point of the analogy is that there are different layers in both the systems [Table 1].

**Table 1:** Analogy between brain and computer system

Computer system	Brain
?	Creativity and Individuality
	(consciousness conscious of itself)
Software applications	Thoughts – induction, inference
Operating System	Memory and Interpretation
BIOS (Basic Input Output System)	Perception Centres
IC (Integrated Circuits)	Neural Pathways
Transistor	Neuron
P–N Junction	Synapse

Can a machine develop ‘consciousness’ and creative operations? Can it become an ‘individual’? The answer is an emphatic ‘No’. The ability to idealise about everything else, and itself, is unique to human consciousness.

Is consciousness a unique set of interconnections in the brain, a kind of flexible circuitboard? Undoubtedly, the basis of all mental activity is physical. But we have access to the higher operations of mind in terms of thought and experience and when we try to approach the system from the lower physical end, the interconnections are not clear. It is very much like a computer. Definitely, all operations of the computer have a basis in the jumping of electrons across the P-N junctions. But as we go upwards to the higher applications, we simply cannot see the operations in terms of electron movements. These higher operations have a grammar of their own which a computer user can master and make use of very effectively without at all understanding what exactly happens in the hardware, as he does all these things.

In the same way, synaptic junctions are the basic points of neuronal activity where most complex processes ultimately originate. Neural pathways are like integrated circuits that form components of the ultimate microprocessor. They create the Basic Input/Output System in terms of various perception centres, the visual cortex being the most well-developed example. As we move higher, we see something like a software similar to the operating system in the form of cognitive structures where all the inputs of the perception centres are integrated to form patterns. These patterns are created with the help of memory and are stored in the memory. They are retrieved later directly for use of the data or indirectly for pattern matching in future processing. The computer uses limited types of signals to create complex systems through the mediation of layers of software. The brain can be modelled in the same way within the broader framework of global workspace theory.

Interestingly, much of this activity is not conscious. The more basic processes are automatic. As more complex operations take place, conscious intervention is necessary. If the processes are stored and rehearsed properly, conscious subroutines tend to become unconscious and automatic.

At the higher level, we have to assume the existence of some kind of Central Executive localised in the well-developed cerebral cortex, probably in the prefrontal area and the frontal lobe. This Central Executive coordinates its activities with various localised centres like Broca’s area, visual cortex, Reticular Activating Formation, etc.

At this higher level, the role of language becomes very important. Language is a code through which most of our thoughts are represented. Language makes it possible for humans to form concepts and relate them to the outside world. Though a lot of mental activity is symbolic and nonverbal, it is through language that we become conscious of our own mental processes.

## Mental Organs

Noam Chomsky proposes that we can think of language as one of the ‘mental organs’ which, in coordination with other mental organs, carries out cognitive processes. It is important to note here that the word organ does not imply that different faculties like language are localised in a particular area of the brain. It is more of a particular alignment of neuronal connections which is flexible. It is more like the software of the operating system. To give a simple working definition: *A mental organ is a set of cognitive operations that operate upon similar type of data in similar ways to create similar type of knowledge in all human beings.* Chomsky deliberately uses the word organ to stress the physical and biological reality of what he is referring to:

In my opinion, the little that we know about these questions suggests that the mind, like the body, is in effect a system of organs - we could call them ‘mental organs’ by analogy - that is to say, highly specific systems organized according to a genetic program that determines their function, their structure, the process of their development, in quite a detailed manner. The particular realization of these fundamental principles naturally depends on their interaction with the environment. If that is correct, the mind is a complex system of interacting faculties - it is constituted of ‘mental organs’ just as specialized and differentiated as those of the body (Chomsky, 2003, p83).

All human beings develop language regardless of conscious training or obvious rewards and punishments. The capacity to process language data and internalise its basic system is innate in every human being. It is the operating system that we inherit, but its development depends upon the richness of the environment. Though higher level uses of language require conscious education, the basic system is easily acquired. The elders around a child do not provide properly tuned data to the child, but his mental organ can form hypothesis, can use induction and differentiation to develop a system capable of creativity that is capable of creating sentences and expressing meaning which it has not heard from anyone. Like any other physical organ, there are distinct milestones in its development with a critical age, after which learning cannot be as effortless. Learning a new language or a new system of pronunciation after puberty is impossible without great conscious effort and motivation. There is some evidence in research on memory by Tulving and Thomson (Gavin, 1998, p57) that we have a distinct processing mechanism for semantic processing which points to a distinct cognitive faculty, the mental organ of ‘language.’

‘Mental organ’ is a concept on a unique plane where physical and nonphysical (call it mental or psychological) come together. It is an interface where the basic software in the BIOS and Operating System is genetically inherited in terms of the hardware. Above this base, learning and environmental factors create higher application software. Human beings are capable of writing their own programmes at the higher level and these programmes are capable of being transmitted to the unconscious hardware where they can become automatised. Of course, it must be recognised that Chomsky introduces the concept as a working hypothesis:

I am not about to propose all this as a new dogma, to replace empiricist doctrine. On the contrary, just as in studying the body, we must simply retain an open mind on this subject. We know a little about a number of cognitive systems, language being the most interesting case at the moment… the important thing is to determine the deeper principles and the detailed structure of various cognitive systems, their modes of interaction, and the general conditions which each system satisfies (Chomsky, 2003, p83).

Mental organs can be better understood in a developmental perspective, that is, study of the way each mental organ (faculty) develops in an individual (keeping aside the evolutionary concerns about the species, at least to begin with).

## Concluding Remarks [See also Figure 1]

**Figure 1:**
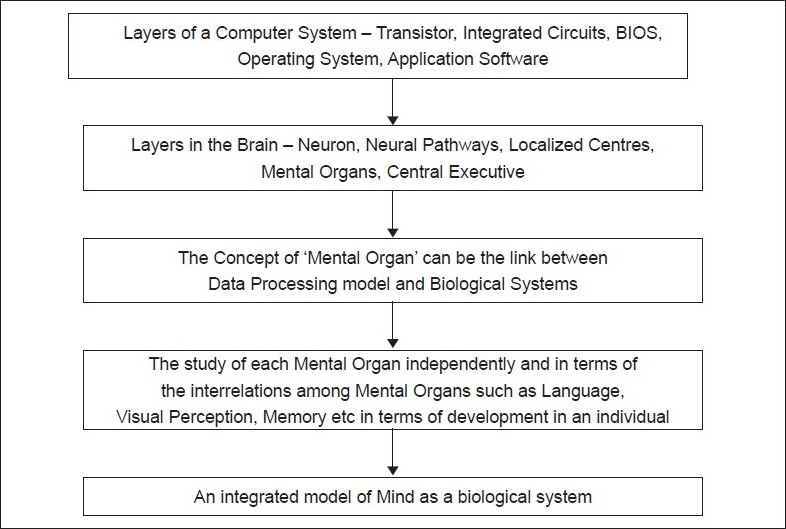
Flowchart of paper

The Central Executive mentioned earlier is nearest to what can be called human consciousness. It is always experienced and understood as individual consciousness where a particular organism has undergone a unique process of development of his mental organs. Chomsky insists upon this biological nature of consciousness while accepting its limitless potential.

Of course, this is on the assumption that the human mind is part of nature, that it is a biological system like the others that we know about but a biological system nevertheless, with its potential scope and its intrinsic limits determined by the very factors that provide its scope. Human reason, on this view, is not the universal instrument that Descartes took it to be but rather a specific biological system (Chomsky, 2003, p66).

Cogito ergo sum.

–Rene Descartes

(I think *therefore* I am.)

Or is it

‘I think *because* I am’?

### Take home message

Data processing and computational models of consciousness can provide important insights into the complex functioning of the human brain. These insights can be put into perspective by using the concept of ‘mental organ,’ which can be further explored, redefined and modified to provide an integrated model of mind, brain and consciousness. The interactions between hardware and software by means of different layers in a computer can be used as a model to explore this system. This may help understand mind as a biological system capable of processing external and internal stimuli in a unique manner.

## Questions That This Paper Raises

1. Is it possible to develop a model architecture of the brain on the basis of the layered structure of the computer?
2. Which are the mental organs that can be studied as distinct systems?
3. Is mental organ like the software applications in a computer?
4. Can the concept of mental organ incorporate both the hardware and software aspects of brain?
5. Can the concept of mental organ be developed in a way totally different than that of Noam Chomsky?

## About the Author

*Manoj Patharkar is Assistant Professor in English at Joshi Bedekar College, Thane. His areas of interest include Grammar, English Language Teaching and English Fiction. He is also keenly interested in interdisciplinary approaches that try to bring together insights from humanities and science.*
